# Mucinous tubular and spindle cell carcinoma of the kidney: A case report and review of the literature

**DOI:** 10.3892/ol.2014.1783

**Published:** 2014-01-07

**Authors:** NAO SUN, YAOWEN FU, YUANTAO WANG, TENGZHENG TIAN, WEI AN, TONG YUAN

**Affiliations:** 1 Department of Urology, First Hospital of Jilin University, Changchun, Jilin 130021, P.R. China; 2 Department of Anesthesiology, First Hospital of Jilin University, Changchun, Jilin 130021, P.R. China

**Keywords:** mucinous tubular and spindle cell carcinoma, immunohistochemistry, kidney neoplasms

## Abstract

Mucinous tubular and spindle cell carcinoma of the kidney (MTSCC-K) is an unusual renal tumor. It is important to increase the recognition of MTSCC-K and improve the level of clinical diagnosis. The current study presents a case of MTSCC-K with clinical, imaging and pathological examination. A 60-year-old female presented to the First Hospital of Jilin University suffering from lumbodorsalgia on the right side for approximately one month, without gross hematuria and fever. Imaging examination by abdominal computed tomography scan revealed a ~6.5×5.0-cm solid mass in the inferior pole of the right kidney. The patient underwent laparoscopic radical resection of the right kidney. Pathological examination showed that the tumor was composed of small, elongated cords or tubules, in a tightly packed arrangement. Myxoid stroma was shown to be interspersed among the tubular cells, and appeared to exhibit slender tubular spindle cell-like structures. Tumor cells were smaller and cube-shaped or oval, with single small eosinophilic nucleoli and low-grade nuclei. Occasionally, necrosis and foam cell infiltration were observed. Myxoid stroma was stained by acidic mucus. Immunohistochemical markers, including CK7, CK19, EMA, Vimentin and P504S (AMACR) showed positive expression in tumoral cells, but the tumoral cells were CD10-negative. The MTSCC-K is a low-grade polymorphic renal epithelial neoplasm, which may be diagnosed by immunohistochemistry. The patients are likely to have an improved prognosis following surgery compared with patients with other renal cell carcinomas.

## Introduction

Mucinous tubular and spindle cell carcinoma of the kidney (MTSCC-K) is a rare renal epithelial tumor, believed to be a type of low-grade malignant tumor. The precise origin is unclear certain researchers have hypothesised that it originates from the loop of Henle or the distal tubule, however the majority of researchers hypothesise that its origin is in the distal tubule (1). MTSCC-K is difficult to pathologically differentiate from high-grade malignant renal tumors, including collecting duct carcinoma and sarcomatoid carcinoma. It was first reported in 1997 by MacLennan *et al* and was known as a ‘low-grade collecting duct carcinoma’ (2). As the number of MTSCC-K cases have increased continuously, this special type of kidney tumor has been described as ‘a unique group of renal neoplasms composed of cytologically low-grade cells organized in tubules and spindled cords and set in an abundant extracellular mucinous matrix’ (3,4). In 2004, the World Health Organization recognized the tumor as a specific entity and officially named it ‘MTSCC-K’ (5). MTSCC-K has a relatively good patient prognosis when compared with other malignant renal tumors (6). The present study analyzed the clinical results of a patient who presented to the First Hospital of Jilin University (Changchun, China) suffering from MTSCC-K, and performed a review of the relevant literature, to increase understanding of the tumor. Additionally the purpose of this study was to raise awareness of this tumor type for clinicians and pathologists in order to decrease the rate of misdiagnosis.

## Case report

### Clinical results

**Figure 1:**
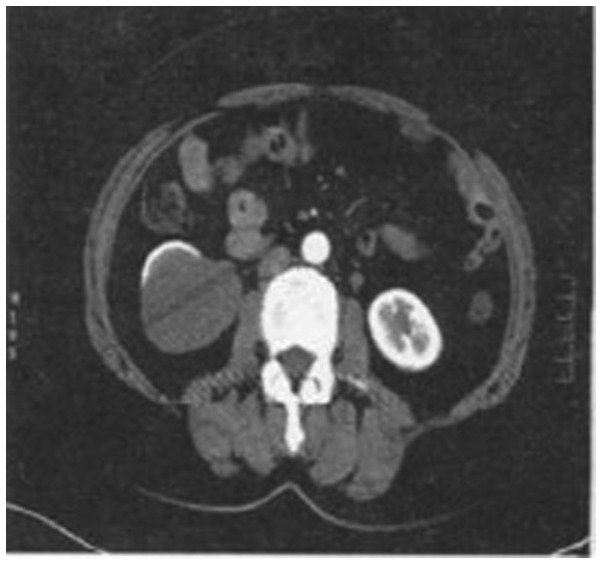
Arterial phase computed tomography. The tumor showed no clear enhancement in this phase.

**Figure 2:**
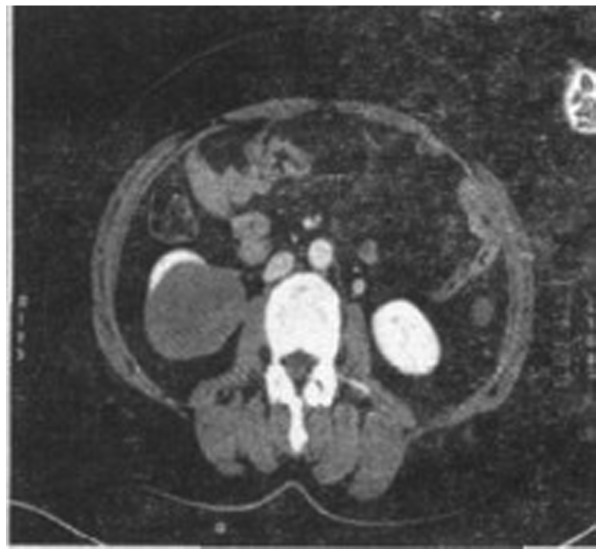
Venous phase computed tomography. The tumor showed marginal uneven enhancement in this phase.

A 60-year-old female presented to the First Hospital of Jilin University suffering from lumbodorsalgia on the right side for approximately one month, without gross hematuria and fever. Laboratory tests revealed no significant abnormalities in renal function, routine blood tests or urine routine. Imaging examination by abdominal computed tomography (CT) scan revealed a ~6.5×5.0-cm solid mass in the inferior pole of the right kidney. The tumor was well-circumscribed and protruding outside the renal contour, and no clear enhancement was identified in the arterial phase (Fig. 1). However, marginal uneven enhancement was observed in the venous phase (Fig. 2). No metastasis was identified to the retroperitoneal lymph node, abdominal organs or lungs. The patient provided written informed consent.

### Surgical procedures

The patient was placed under general anesthesia and underwent laparoscopic radical resection of the right kidney (surgical excision of the right kidney, perirenal fat, a section of the ureter over and the right adrenal gland).

### Macroscopy

Dissection of the specimen revealed that the tumor was well-circumscribed, solid and off-white, measuring ~7.0×6.5×6.5 cm. No areas of hemorrhage or necrosis were identified in the tumor. In addition, no invasion of the renal pelvis, renal sinus or perirenal fat was identified.

### Microscopy

**Figure 3:**
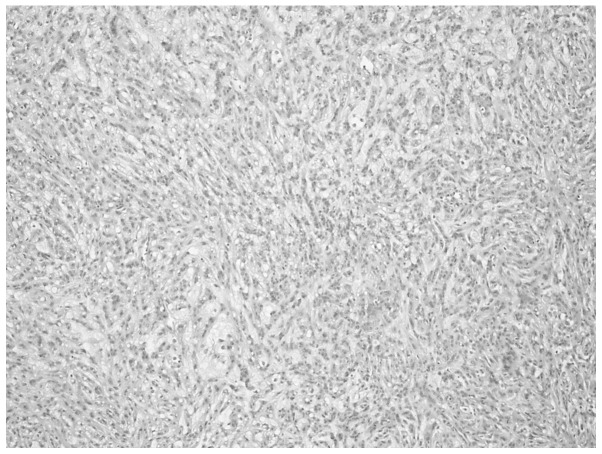
Tumor was composed of small, elongated cords or tubules, in a tightly packed arrangement (hematoxylin and eosin; magnification, ×10).

**Figure 4:**
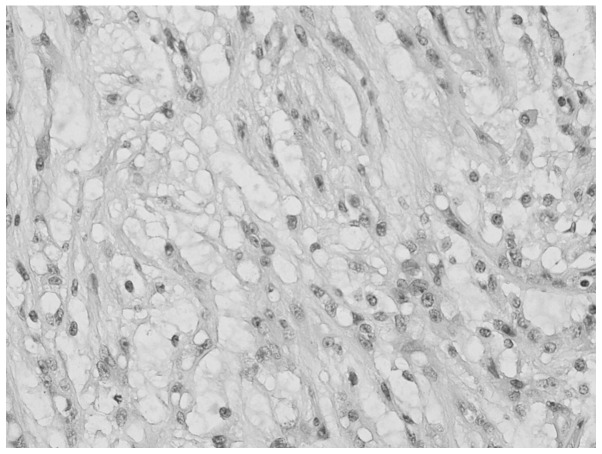
Myxoid stroma was interspersed among the tubular cells (hematoxylin and eosin; magnification, ×40).

**Figure 5:**
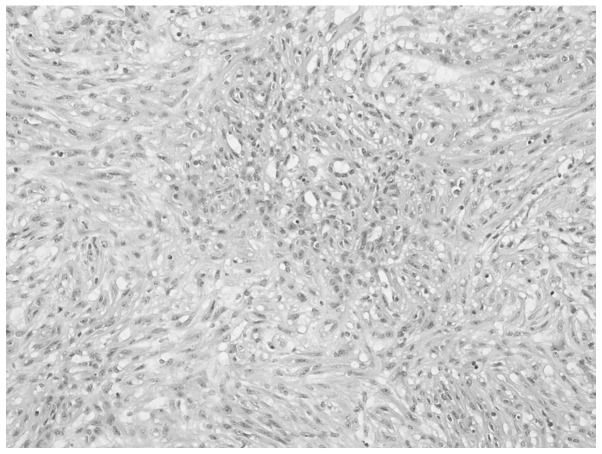
Slender tubular spindle cell-like structures (hematoxylin and eosin; magnification, ×20).

**Figure 6:**
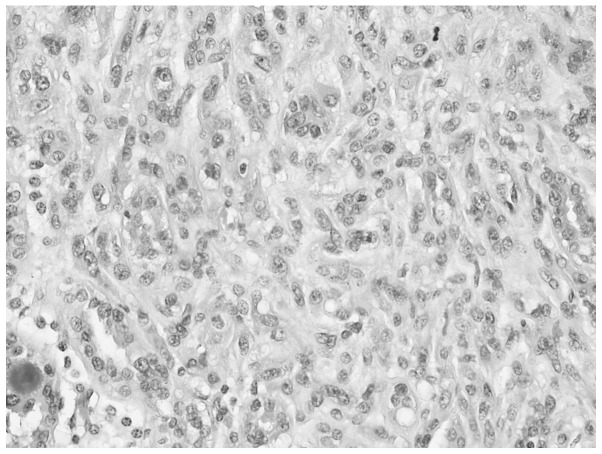
Tumor cells were smaller and cube-shaped or oval, with single small eosinophilic nucleoli and low-grade nuclei (hematoxylin and eosin; magnification, ×40).

**Figure 7:**
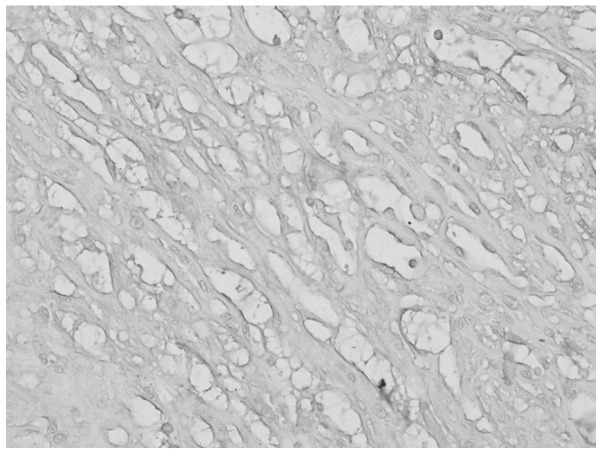
Myxoid stromal staining by acidic mucus (alcian blue; magnification, ×40).

The tumor was composed of small, elongated cords or tubules, in a tightly packed arrangement (Fig. 3). Myxoid stroma was shown to be interspersed among the tubular cells (Fig. 4), and appeared to exhibit slender tubular spindle cell-like structures (Fig. 5). Tumor cells were smaller and cubic-shaped or oval, with single small eosinophilic nucleoli and low-grade nuclei (Fig. 6). Occasionally, necrosis and foam cell infiltration were identified. The myxoid stroma was stained by acidic mucus (Fig. 7).

### Immunohistochemistry

**Figure 8:**
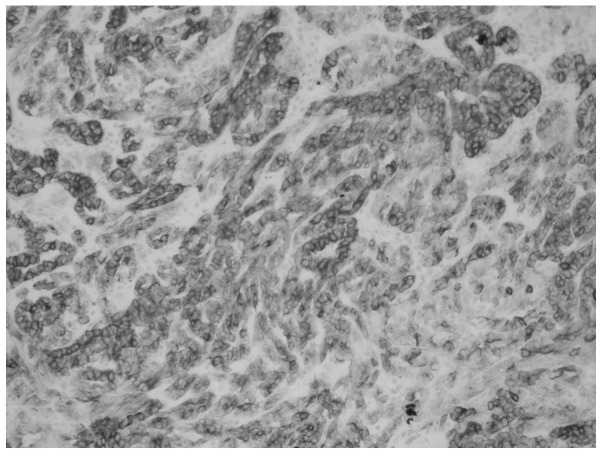
CK7 showed positive expression in tumoral cells (magnification, ×20).

**Figure 9:**
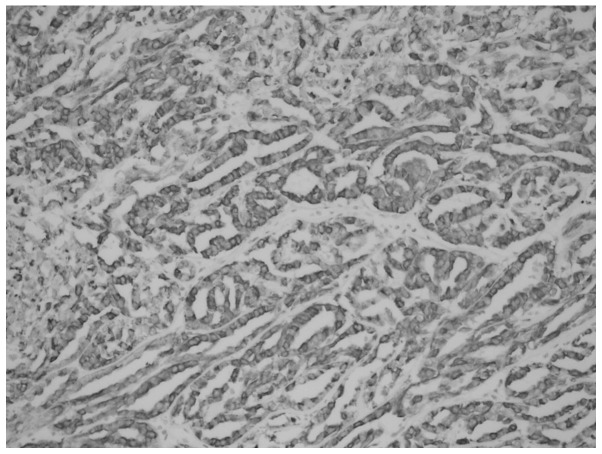
CK19 showed positive expression in tumoral cells (magnification, ×10).

**Figure 10:**
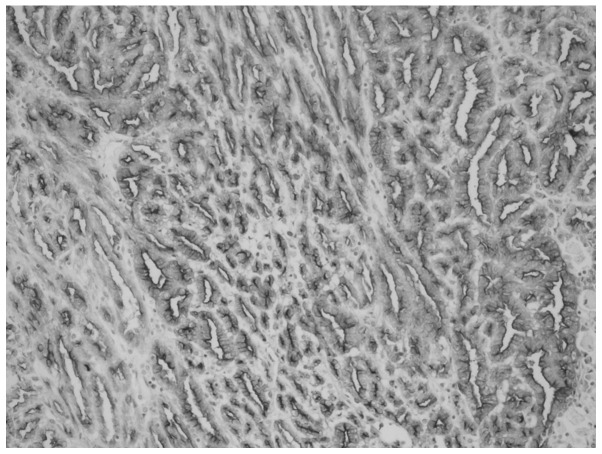
EMA showed positive expression in tumoral cells (magnification, ×10).

CK7 (Fig. 8), CK19 (Fig. 9), EMA (Fig. 10), Vimentin and P504S (AMACR) showed positive expression in tumoral cells, but the tumoral cells were CD10-negative.

### Pathological results

The tumor was well-circumscribed, measuring ~7.0×6.5×6.5 cm. Invasion of the renal pelvis, renal sinus or perirenal fat was not observed. Under the microscope (BX53, Olympus, Tokyo, Japan), the tumor observed to be composed of small, elongated cords or tubules. Myxoid stroma was demonstrated to be interspersed among the tubular cells and appeared to exhibit slender tubular spindle cell-like structures, and was stained by acidic mucus. In terms of immunohistochemical staining, CK7, CK19 and AMACR had a positive expression in tumoral cells, while CD10 was negative in the tumorous cells. From these observations the tumor was determined to be low grade T1bN0 MTSCC-K.

## Discussion

To date, ~100 MTSCC-K cases have been reported worldwide (6). MTSCC-K has a wide age distribution, with an age range between 17 and 82 years and a mean age of 53 years. The female incidence is approximately three- or four-fold that of males (3,6–8). Typically, the majority of patients present with asymptomatic masses, often found incidentally by ultrasound (9,10). In a few cases, the patient may present with flank pain or hematuria (9).

One of the main features of MTSCC-K is the good prognosis. However, the clinical symptoms and imaging characteristics of the MTSCC-K are similar to the more common renal cell carcinoma (RCC). Therefore, a clear preoperative diagnosis becomes more difficult. Previously, it has been reported (11) that on renal magnetic resonance imaging scans for the tumor, the T2 signal intensity ratio is 0.96 (intermediate between papillary, 0.67 and clear cell carcinoma, 1.41). Additionally, T1 images show a signal similar to that of a normal renal cortex and postcontrast T1 VIBE images demonstrate poor early phase enhancement. The current case underwent abdominal CT scan prior to surgery and no clear enhancement was identified in the arterial phase. However, marginal uneven enhancement was identified in the venous phase. This change is consistent with the previous literature and may provide references for the preoperative clinical diagnosis of MTSCC-K. In addition, it has been reported that fine needle aspiration biopsy may be diagnostic of MTSCC-K (12), which may aid to improve preoperative diagnosis rates.

Previously, Fine *et al* (3) described the histological types of MTSCC-K and divided them into the following two categories: Classic, abundance of extracellular mucin stroma; and mucin-poor, little to no extracellular mucin. In addition, the unusual organizational characteristics associated with MTSCC-K are as follows: Papillary clear cell structure, necrosis, ectopic bone or grit-like calcospherites and neuroendocrine differentiation has also been reported (13).

In cytogenetic studies, genetic abnormalities found in MTSCC-K cells are monosomies in chromosomes 1, 4, 6, 8 and 13, and total or partial trisomies of chromosomes 7, 11, 16 and 17 (3). Simultaneously, the immunophenotype of MTSCC-K tumor cells demonstrates a complex mode, including epithelial markers (CK19, CK7 and AE1/AE3) and distal convoluted tubule markers (EMACK19 and E-cadherin).

Since the incidence ratio of papillary RCC is second to clear RCC and is also a relatively common pathological type of RCC, the tubular architecture showing focal papillae are clear clues pointing to MTSCC-K. Therefore, it is important to differentially diagnose papillary RCC and MTSCC-K. Associated studies (13) have previously reported on a few of the immunohistochemical markers between MTSCC-K cells and papillary RCC cells. By comparison, the immunoreactivity in MTSCC-K was as follows: AMACR, 93%; CK7, 81%; EMA, 95%; RCC Ma, 7%; CD10, 15%; HMWK, 15%; and c-kit, 5%. While in papillary RCC, the immunoreactivity was as follows: AMACR, 95%; CK7, 65%; EMA, 88%; RCC Ma, 25%; CD10, 80%; HWMK, 15%; and c-kit, 18%. AMACR was once the specific immunohistochemical marker of papillary RCC, but high expression of AMACR has also been confirmed in MTSCC-K cells. By immunohistochemistry, in addition to the rate of CD10 expression differences existing in the two tumor cells, other markers have shown similarities. Therefore, identification of the two tumors relies mainly on pathological and karyotype analysis. Papillary RCC does not have an extracellular matrix; however, chromosomal gains, particularly in chromosomes 7 and 17, and loss of chromosome Y characterize papillary RCC (4).

Overall, MTSCC-K exhibits a lower malignant degree and an improved prognosis compared with other types of RCC (14). Generally, radical nephrectomy is the best treatment and no additional treatment is required following surgery. The tumor is typically of low pathological stage at the time of excision and it is extremely rare for cases to present with metastases to lymph nodes and other organs at the time of diagnosis (15). To date, an extremely small number of reported cases have presented with distant metastasis and no tumor-related mortality has been reported (6). However, MTSCC-K, as a type of renal cancer, requires close follow-up after surgery.
